# Durable response of glioblastoma to adjuvant therapy consisting of temozolomide and a weekly dose of AMD3100 (plerixafor), a CXCR4 inhibitor, together with lapatinib, metformin and niacinamide

**DOI:** 10.18632/oncoscience.311

**Published:** 2016-06-11

**Authors:** Adan Rios, Sigmund H. Hsu, Angel Blanco, Jamie Buryanek, Arthur L. Day, Mary F. McGuire, Robert E. Brown

**Affiliations:** ^1^ Division of Oncology at UTHealth McGovern Medical School, Houston, TX, USA; ^2^ Department of Neurosurgery at UTHealth McGovern Medical School, Houston, TX, USA; ^3^ Memorial Hermann Hospital, Texas Medical Center, Houston, TX, USA; ^4^ Department of Pathology and Laboratory Medicine at UTHealth McGovern Medical School, Houston, TX, USA; ^5^ Adjunct Faculty, Mathematics & Computer Science at University of St. Thomas-Houston, Houston, TX, USA

**Keywords:** glioblastoma, morphoproteomics, biomedical analytics, plerixafor, preventative and targeted therapy

## Abstract

Glioblastoma multiforme (GBM) is a CNS (central nervous system) malignancy with a low cure rate. Median time to progression after standard treatment is 7 months and median overall survival is 15 months [1]. Post-treatment vasculogenesis promoted by recruitment of bone marrow derived cells (BMDCs, CD11b+ myelomonocytes) is one of main mechanisms of GBM resistance to initial chemoradiotherapy treatment [2]. Local secretion of SDF-1, cognate ligand of BMDCs CXCR4 receptors attracts BMDCs to the post-radiation tumor site.[3]. This SDF-1 hypoxia-dependent effect can be blocked by AMD3100 (plerixafor) [4]. We report a GBM case treated after chemo- radiotherapy with plerixafor and a combination of an mTOR, a Sirt1 and an EGFRvIII inhibitor. After one year temozolomide and the EGFRvIII inhibitor were stopped. Plerixafor, and the MTOR and Sirt-1 inhibitors were continued. He is in clinical and radiologic remission 30 months from the initiation of his adjuvant treatment. To our knowledge, this is the first report of a patient treated for over two years with a CXCR4 inhibitor (plerixafor), as part of his adjuvant treatment. We believe there is sufficient experimental evidence to consider AMD3100 (plerixafor) part of the adjuvant treatment of GBM.

**Significance:**

The adjuvant inhibition of GBM vasculogenesis(a process different from local angiogenesis) by specifically blocking the migration of BMDCs to the primary tumor site with inhibitors of the CXCR4/SDF-1 axis represents a potential novel therapeutic approach to GBM. There is significant pre-clinical evidence and validation for its use as demonstrated in a patient derived tumor xenograft model of GBM. Together with other specific anti-tumoral therapies, the active inhibition of vasculogenesis in the adjuvant treatment of GBM is deserving of further exploration.

## CASE REPORT

### Patient clinical course and treatment history

**Figure 1:**
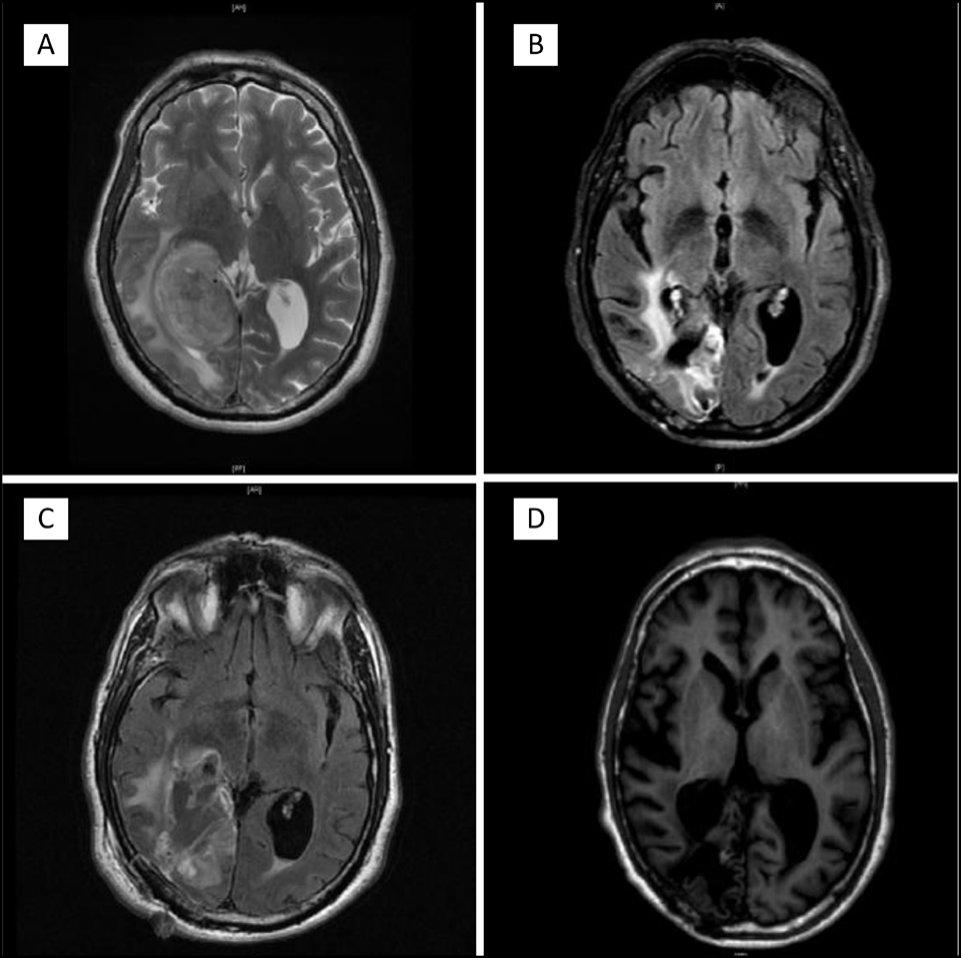
A. Image of pre surgical MRI of September 09 2013 showing within the posterior medial right temporal and occipital lobes a 3.8 × 5.8 × 4.7 cm. mass. The mass had peripheral enhancement and effacement of the atrium of the right lateral ventricle, displaced laterally. B. Post- surgery image of September 21 2013, showing interval right parietal craniotomy with significant debulking of right temporal occipital tumor. There was a large area of restricted diffusion along the margins of the resection cavity in the right occipital lobe. There was expected postoperative enhancement of the margins of the resection cavity and along the operative tract. C. At one year post- surgical intervention and completion of temozolomide adjuvant treatment, there were evolving intrinsic T1 hyper-intensities along the margins of the resection cavity in the right parietal lobe. There was improvement in the enhancement along the margins of the operative tract and section cavity. No new areas of enhancement, hydrocephalus or midline shift. D. At thirty months follow up there were post-operative changes with no new enhancing lesions, no hydrocephalus or midline shift. The patient is neurologically functional and with a ECOG PS* of 0. *Eastern Cooperative Group Performance Status.

A 66-year-old male patient began to experience vision difficulties, developed a persistent right fronto- temporal headache and memory loss for recent events. On September 3, 2013, after an evaluation, a CT of the brain was done in Panama City, Panama. The CT demonstrated a right temporal occipital tumor with significant mass effect. The patient was started on oral dexamethasone and opted to seek further management in Houston, Texas. He was evaluated at our institution by the neurosurgery team and found to have a dense left homonymous hemianopsia. A brain MRI confirmed the presence of a medial right temporo-occipital lobe mass measuring 3.8 × 5.8 × 4.7 cm^3^ with peripheral enhancement and centrally decreased T1 signal. There was increased T2 signal throughout the white matter, with effacement of the atrium and right lateral ventricle and with lateral displacement (Figure 1A). In addition, there was superior medial displacement of the adjacent posterior cerebral artery. In September 19, 2013, he underwent a right parieto-occipital craniotomy with maximal tumor removal (Figure 1B). Surgical pathology revealed a GBM WHO grade IV. Gene sequencing analysis as well as morphoproteomic analysis of the tumor was requested. Four weeks after the surgery treatment was initiated with chemo-radiotherapy. He received a total of 60 Gray (Gy) in 30 fractions with a concurrent dose of temozolomide of 75mg/m^2^ BSA daily for 42 days through the duration of the radiotherapy. Four weeks after completion of the chemo-radiotherapy he was started on adjuvant treatment with a combination of temozolomide and plerixafor. The temozolomide dose was 150mg/m^2^ BSA daily for five days the first month of treatment and subsequently escalated to 200mg/m^2^ BSA daily for five days monthly. Plerixafor was given subcutaneously at a dose of 0.24mg/Kg of body weight once a week. In addition he received a daily dose of lapatinib, niacinamide and metformin. At the completion of twelve months the temozolomide was stopped. He has continued the administration of the weekly plerixafor and daily niacinamide and metformin. Since completion of his concurrent chemo-radiotherapy and initiation of his adjuvant treatment there has been progressive stabilization of his clinical condition and of his brain MRI images (Figure 1 C and D) with no evidence of recurrence of his tumor. His vision has improved with the assistance of special lenses and he has returned to work. Adverse events experienced during this treatment include a skin rash with diarrhea associated to the lapatinib, infrequent periods of thrombocytopenia during the temozolomide period of treatment and a right popliteal venous thrombosis treated temporarily with enoxaparin. Initially he experienced several months of depression requiring the administration of an antidepressant. His initial weight loss improved once the temozolomide was discontinued and with the assistance of a short course of anabolic steroids and physical therapy.

### Molecular analyses

**Figure 2:**
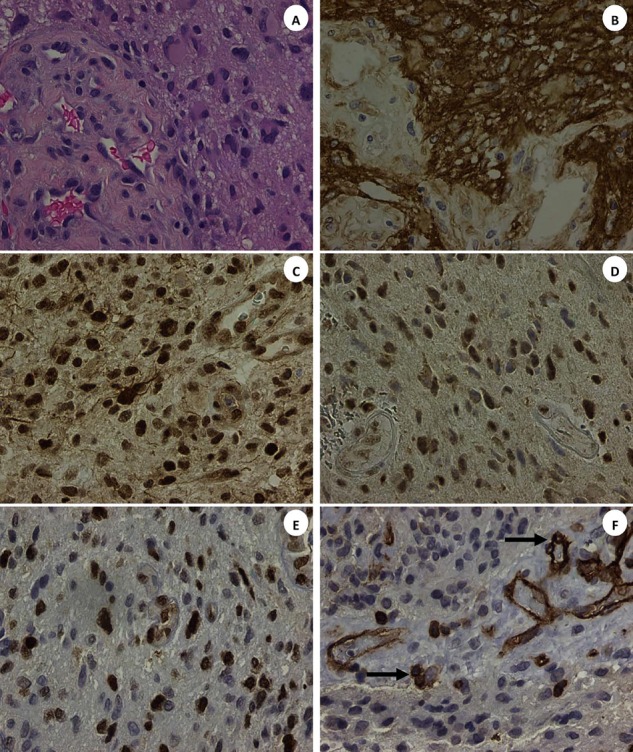
The patient's glioblastoma multiforme (GBM) with: H&E stained section showing tumor cells with adjacent angiogenic focus (Frame A); high expression of epidermal growth factor receptor, total and vIII on the plasmalemmal aspect of the tumor cells(Frame B); nuclear compartmentalization of phosphorylated (p ) mTOR (Ser 2448) (Frame C); variable expression of Sirt1 Frame D) and EZH2 (Frame E) in the nuclei of the tumor cells; and CXCR4 on the endothelial cells including areas of tumoral angiogenesis along with occasional scattered mononuclear cells in the adjacent tissues (Frame F, see arrows) There is also an associated expression of vascular endothelial growth factor (VEGF)-A in the tumor cells in the angiogenic region. PKC-alpha, p-Akt (Ser 473) and VEGF-A are not depicted. (DAB[3,3′-diaminobenzidine] brow**n** chromogenic signal; original magnifications x400 for Frames A-F).

The hematoxylin-eosin stained section of the patient's GBM revealed an infiltrating, cellular glial neoplasm comprised of an astrocyte-like and tumor cells. Mitotic index was estimated 14 per 10 high power fields. Morphoproteomic analysis [5] demonstrated the following: epidermal growth factor receptor (EGFR total and EGFRvIII ) was expressed at 2-3+ (scale 0-3+) on the plasmalemmal surface of the malignant glial cells; cytoplasmic and plasmalemmal expression of protein kinase C (PKC)-alpha; activation of downstream effectors and pathways of convergence evidenced by the constitutive activation of the mammalian target of rapamycin (mTOR)/Akt pathway with predominant nuclear compartmentalization of p-mTOR (Ser 2448) and of p-Akt (Ser 473), up to 3+ signal intensity, for both, indicating dominance of the mTORC2 component of the pathway[6-8]; Sirt1 (silent mating type information regulation 2 homolog 1), an NAD+ histone deacetylase, was found to have variable nuclear positivity up to 2+ in tumor cells; and enhancement of Zeste homolog 2 (EZH2), an histone methyltransferase, was expressed up to 3+ in the majority of tumoral nuclei in some regions(EZH2 has the potential to block differentiation, promote proliferation, and promote tumorigenesis by methylating tumor suppressor genes. It can work collaboratively with Sirt1 in promoting tumorigenesis [9]). In general there was strong expression of the C-X-C chemokine receptor type 4 (CXCR4) on the patient's tumor endothelial cells including areas of tumoral angiogenesis along with occasional scattered mononuclear cells in the adjacent tissues. The tumor cells per se were negative to weakly positive (±) but only focally. These are illustrated in Figure 2 A-F. Correspondingly, in the same regions of tumoral angiogenesis, there was an associated over-expression of vascular endothelial growth factor (VEGF)-A. The gene sequencing study (Foundation One, Boston, Mass.) revealed an unambiguous amplification of *EGFRvIII* and suggested an equivocal amplification of the *AKT*3 gene.

### Biomedical analytics

**Figure 3:**
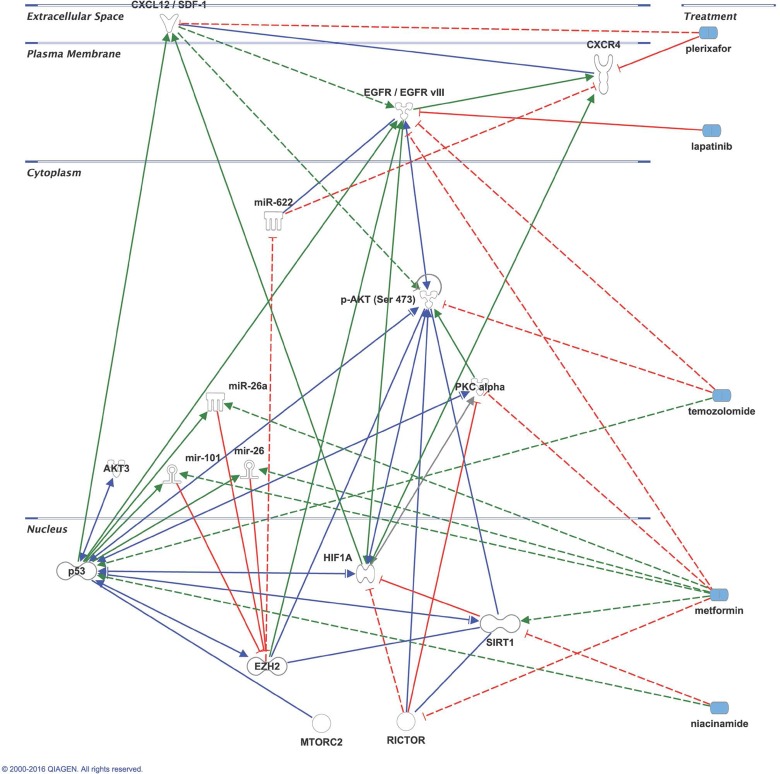
Key interactions modulated by potential pharmacogenomic agents in glioblastoma. Dashed lines: indirect interactions; Red coloration/t-bar: downregulation; Green coloration/arrow: upregulation; Blue coloration/arrow: dual- directional regulation or binding.

The integration of the morphoproteomic and genomic findings with the pharmacogenomic and targeted therapeutic implications of temozolomide, plerixafor, lapatinib, metformin, niacinamide in this context are addressed in the pathway analysis provided by biomedical analytics (Figure 3).

## DISCUSSION

The current standard of care of GBM includes the use of maximal surgical tumor removal followed by combination chemo-radiotherapy with temozolomide administered daily through the duration of the radiotherapy. Adjuvant temozolomide is then given in five days cycles once a month for six cycles. An alternative schedule of twelve cycles for patients tolerating the treatment is commonly used as it was the case of our patient [10]. Despite this aggressive treatment the median times to progression and of survival are 7 and 15 months respectively [1]. Our patient has exceeded both and appears from every clinical and radiological indication that his durable response may extend even further.

Recent experimental evidence indicates that one of the most important mechanisms for relapse of GBMs is the re-colonization of the treated tumor bed by a significant influx of bone marrow derived MMP-9 expressing CD11b+ myelomonocytic cells (BMDCs) [2]. These cells are responsible for the initiation of vasculogenesis, restoring the radiation damaged tumor vasculature and rescuing the surviving tumor cells [11]. This secondary vasculogenesis, different from primary tumor angiogenesis, is the consequence of the destruction of the endothelial cells of the primary tumor by the initial treatment inhibiting local angiogenesis with an increased production of hypoxia-dependent HIF-1. This results in an increased secretion of SDF-1 (CXCL12) by the tumor and stromal tumor-bed cells [12]. SDF-1 or CXCL12 is the cognate ligand of the CXCR4 receptor [13]. This causes an increased mobilization of bone marrow derived cells BMDCs, specifically, CD11b+ myelomonocytes and its retention in the tumor bed [14] allowing the initiation of tumor recurrence through the process of vasculogenesis.

Plerixafor is a bycyclam molecule which is a specific reversible blocker of CXCR4 receptors [15]. Initially developed as a potential blocker of HIV entry into susceptible cells, plerixafor is currently used in combination with granulocyte-colony stimulating factor (G-CSF) as a hematopoietic stem cell mobilizer. [16]. Blocking the CXCR4 receptors with AMD3100 (plerixafor) has demonstrated prevention of tumor recurrence in orthotopic xenografts of glioblastoma in a mouse model [2]. Importantly, in this experimental xenograft model, tumors continued to shrink and did not recur even long after the exposure to plerixafor had ceased. This feature observed in this particular model, was of great significance in deciding the dose and schedule of administration of plerixafor to our patient who at 30 months of initiating his adjuvant treatment continues to receive a weekly dose of it. With the schedule of a single weekly dose of plerixafor there were no obvious clinical or laboratory adverse effects noted in our patient that could be directly attributed to the administration of plerixafor. Clinical studies of plerixafor for the indication of HSC mobilization administered daily for a minimum of four days and in others up to three weeks of daily administration have shown that plerixafor is overall well tolerated. In different clinical studies the most common adverse events associated to plerixafor administration have been observed across the board in only 50% of patients. They include nausea (35%), vomiting (15%), abdominal pain (5%), myalgia (25%) and headache (10%), none of them over grade 2 of CTCAE vs4. [17, 18]. The phenotypic composition of the peripheral blood immediately after the administration of a single dose of plerixafor in our patient has not been studied. Gene sequencing and morphoproteomic studies prospectively suggested that there were other metabolic pathways in the tumor of our patient potentially actionable by specific agents such as lapatinib, metformin and niacinamide. The tumor was found to have by both, morphoproteomic analysis and gene sequencing amplification of *EGFR/EGFRvIII* known to be present in 45% of GBM tumors[15]. This led us to the selection of lapatinib as one of the components of his adjuvant regimen. Lapatinib, a tyrosine kinase inhibitor targets the inactive conformation of EGFR[16]. Our patient was started on a daily dose of lapatinib of 1000mg daily for two weeks every two weeks which he took initially for three months. However, when reports in the literature suggested that daily dosing of lapatinib are not sufficient to achieve a high intratumoral concentration for brain tumors and were proven ineffective in the treatment of recurrent GBM [16] he was switched to a higher dosage of lapatinib given in weekly pulses. He received 2500mg of lapatinib twice a day, for 2 consecutive days per week in combination with the adjuvant TMZ [10] and the other two components of the adjuvant regimen (metformin and niacinamide ) until twelve months of adjuvant temozolomide were completed. While the gene sequencing studies showed an equivocal amplification of AKT3, the morphoproteomic studies revealed a constitutive activation of the mammalian target of rapamycin (mTOR)/Akt pathway with predominant nuclear compartmentalization of p-mTOR (Ser 2448) and of p-Akt (Ser 473), indicating that the mTORC2 component of the pathway was dominant[6-8]. In GBM cell lines, activation of mTORC2 has been found to correlate positively with cell proliferation and motility, mediated in part by PKC-alpha, also expressed in our patient and in accordance with the interpretation of mTORC2 predominance [17]. Silencing of both, EGFR and rictor in GBM has been associated with complete tumor regression when combined with chemotherapy in an orthotopic GBM model [18]. Metformin is known to inhibit both, EGFR and rictor in addition to inhibition of PKC-alpha mediated tumor migration and invasion [19]. Thus, we gradually started metformin at the initiation of his adjuvant treatment at a dose of 500mg a day and escalated to a dose of 1000mg twice a day over a period of three weeks with the patient remaining on metformin until now. Finally, in the morphoproteomic analysis, Sirt1 had variable nuclear positivity in the majority of tumor cells. In GBM, the tumorigenic properties of Sirt1 may be mediated by downstream activation of PI3k/AKT signalling [20], also observed in our patient and thus becoming part of a pathway of convergence. For this reason, at the initiation of his adjuvant treatment, we added a Sirt1 inhibitor, niacinamide, at a dose of 60mg/Kg a day, which is known to inhibit Sirt1 activity in pre- clinical and clinical models of cancer with minimal or no clinical toxicity [21]. As a result of our confirmation that EZH2 was highly expressed in his tumoral nuclei using morphoproteomics, it is noteworthy that EZH2 mediated loss of miR-622 can result in CXCR4 activation [22]. Metformin, via the upregulation of miR-26a and miR-101 downregulates EZH2 [9, 23] and would seem to counteract this effect of CXCR4 activation by EZH2 (see Biomedical Analytics, Figure 3). The blocking of CXCR4 by plerixafor may also have a role in increasing T cell-mediated antitumor immune response, as seen in an immunocompetent animal model.

**Figure 4:**
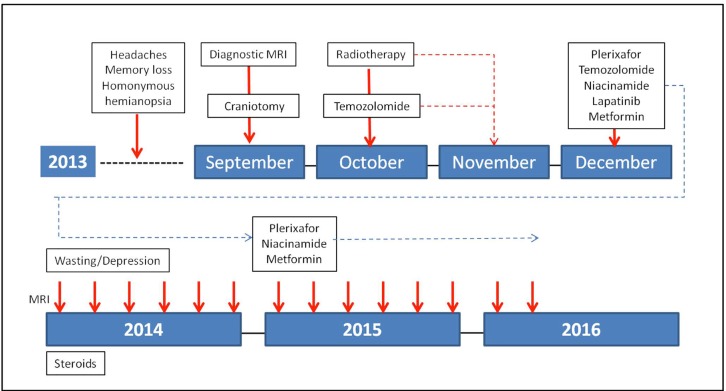
Treatment course from diagnosis until present. Plerixafor was initiated at a dose of 0.24mg/Kg s.c. once a week together with the initiation of the adjuvant treatment with temozolomide, lapatinib, niacinamide, and metformin. It has been given continuously since then. Lapatinib and temozolomide were discontinued after completion of one year of adjuvant treatment. During the last six months of the first year of adjuvant treatment, the patient experienced weight loss, a reactive episode of depression and a superficial phlebitis of the right leg. These symptoms required and resolved after a short course of anabolic steroids, an anti-depressant and anti-coagulation with low molecular weight heparin. After the first year of adjuvant treatment he has continued on treatment only with plerixafor, niacinamide and metformin. His ECOG PS is 0.

On completion of the first twelve months of adjuvant treatment temozolomide and lapatinib were discontinued. At 30 months, he continues to receive treatment with a weekly dose of plerixafor and on metformin and niacinamide at the initially prescribed doses without clinical or radiological evidence of relapse and an excellent quality of life (Figure 4). We recognize the inherent limitations to the report of one case. However, given the well-established natural history of GBM, the unusual duration of this response merits consideration. To our knowledge, this is the first patient treated with an adjuvant program involving a combination of anti- tumoral agents (chemotherapy plus inhibitors of metabolic pathways) and more importantly an agent that specifically inhibits vasculogenesis. We believe that this approach to the adjuvant treatment of GBM is deserving of further clinical exploration.

## MATERIALS AND METHODS

Treatment, obtaining informed consent and molecular analysis were performed in a patient with GBM in accordance with the UT at Houston Institutional Review Board (IRB).

Molecular analysis was performed (RE Brown's Consultative Proteomics Laboratory which is CLIA certified and CAP accredited) to analyse tumor signatures.

### Immunohistochemistry and morphoproteomics

The use of bright field microscopy and immunohistochemistry directed against various protein analytes can better define the biology of GBM process and the pathogenic occurrences that may be responsible for its development, chemoradioresistance, and propensity to recur. That is the application of morphoproteomics [5]. Immunohistochemical probes were applied against the following protein analytes in unstained sections of the patient's original diagnostic biopsy read as GBM: Epidermal growth factor receptor (EGFR PharmDx, total and vIII; DakoCytomation, Carpentaria, CA ); protein kinase C(PKC)-alpha ( Santa Cruz Biotechnology Inc, Santa Cruz, CA ); mammalian target of rapamycin (mTOR), phosphorylated on serine 2448 (Cell Signaling Technology Inc., Danvers, MA); Akt, phosphorylated on serine 473 (Cell Signaling Technology Inc. ); silent mating type information regulation 2 homolog (SIRT)1( Abcam Inc, Cambridge, MA);enhancer of Zeste homolog 2 (EZH2; Cell Signaling Technology, Inc. ); CXCR4 chemokine receptor ( Abcam Inc); and vascular endothelial growth factor (VEGF)-A (DakoCytomation). The level of expression of the analytes was graded on a 0 to 3+ scale using bright field microscopy and based on signal intensity indicated by a 3,3′-diaminobenzidine tetrahydrochloride (DAB) chromogenic (brown) signal. Positive and negative external controls were run concurrently and noted to react appropriately. Internal controls to include contiguous, non-neoplastic elements of the patient's specimen confirmed the immunopositivity of the patient's tumor cells. A negative control of the tumor, minus the primary antibody, reacted appropriately.

### Biomedical analytics

Biomedical analytics develops and applies methods from mathematics and computer science to gain insights into biological processes based on personalized data and published biomedical research. In this study, biomedical analytics integrated the morphoproteomic analysis of a patient's glioblastoma with the known effects and interactions of temozolomide, plerixafor, lapatinib, metformin and niacinamide on signal transduction pathways and prevention of CXCR4 bone marrow derived cell vasculogenic repair to account for the patient's extended remission. The patient's data were normalized and weighted by an algorithm customized for the pathologist of record. The resulting score for each analyte was entered, along with its UNIPROT ID, into Ingenuity Pathway Analysis (IPA, http://www.ingenuity.com). Pathway networks and their interactions with the proposed therapies were evoked based on existing IPA data. From these graphs and additional data mining of the National Library of Medicine's MEDLINE data base, a single network model was constructed using IPA Pathway Designer to represent the key immune modulation and adaptive responses in the signal transduction processes.

For key of symbols interpretation of Fig 3 please refer to: http://ingenuity.force.com/ipa/articles/Feature_Description/Legend
